# *Achillea millefolium* ameliorates doxorubicin-induced renal injury via inhibition of oxidative stress and inflammation in rats

**DOI:** 10.55730/1300-0144.5922

**Published:** 2024-09-18

**Authors:** Mohammed SHAIEA, Yiming DONG, Saleh ALOMAISI, Hassan AL-MAHBASHI, Guozhong ZHANG, Chuan WANG

**Affiliations:** 1 Department of Pharmacology, the Key Laboratory of New Drug Pharmacology and Toxicology, Hebei Medical University, Shijiazhuang, China; 2 Department of Anatomy and Embryology, Faculty of Veterinary Medicine, Sana’a University, Sana’a, Yemen; 3 Department of Forensic Medicine and Clinical Toxicology, Faculty of Medicine and Health Sciences, Sana’a University, Sana’a, Yemen; 4 Hebei Province Laboratory of Experimental Animal, Shijiazhuang, China

**Keywords:** *Achillea millefolium*, doxorubicin, oxidative stress, inflammation, nephrotoxicity

## Abstract

**Background/aim:**

Doxorubicin (Dox) is a potent anticancer medication. However, due to nephrotoxicity, its clinical application is restricted. *Achillea millefolium* (AM) is a plant used in traditional medicine to treat several conditions, including kidney disorders. The aim of this work was to investigate the preventative properties of AM extract (AME) and their mechanisms against nephrotoxicity caused by Dox in rats.

**Materials and methods:**

The rats were assigned randomly to six groups, including a control group, Dox group (5 mg/kg/week via i.p. for 4 weeks), two groups receiving AME (100 or 200 mg/kg, orally for 28 days), and the last two groups receiving Dox + AME (100 or 200 mg/kg, orally for 4 weeks). After the treatment period concluded, samples of blood and renal tissue were collected for analysis. Serum creatinine, urea, and uric acid levels were used to determine nephrotoxicity biochemically. In renal tissue samples, superoxide dismutase (SOD), catalase, glutathione (GSH), glutathione peroxidase (GPx), total antioxidant capacity (TAC), nitric oxide (NOx), tumor necrosis factor-alpha (TNF-α), interleukin-1 beta (IL-1β), and nuclear factor kappa B (NF-κB) were measured. Histopathological analysis of the kidneys was also performed.

**Results:**

Dox caused a considerable increase in kidney function parameters and the occurrence of histological changes, which were significantly reversed by AME treatment. Mechanistically, Dox caused renal oxidative stress by raising malondialdehyde and NOx levels while lowering SOD, GSH, GPx, and TAC. It also caused inflammation via the stimulation of proinflammatory cytokines in renal tissues. Conversely, the treatment of AME mitigated Dox-evoked abnormalities in the above-mentioned tests.

**Conclusion:**

AME could protect against nephrotoxicity caused by Dox by reducing oxidative stress, stimulating antioxidant mechanisms, and suppressing proinflammatory cytokines, suggesting that AME may be useful as an adjuvant therapy for Dox-induced nephrotoxicity.

## 1. Introduction

Chemotherapy has become the most widely utilized method for the treatment of cancer. It enhances cancer patients’ quality of life and provides hope for cancer remission [1]. Despite their high efficacy, the most potent chemotherapy medications have side effects by destroying both malignant and normal cells [2,3]. *Streptomyces peucetius* generates daunorubicin, an antibiotic that has been modified genetically to make adriamycin, commonly known as doxorubicin (Dox) [4]. Dox, a potent chemotherapy drug known as an anthracycline medication, has been extensively prescribed for solid cancers and hematological tumors since its discovery [3,5,6]. However, the medical application of Dox is restricted due to its deleterious effects on main organs such as the liver, kidney, and heart. Renal toxicity is one of its most prevalent adverse effects and has been associated with a high risk of morbidity as well as mortality. Nephrotoxicity occurs in about 60% of chemotherapy-treated cancer patients, which restricts the curative efficacy of Dox [3,5–7]. It accumulates in the kidneys and causes direct renal damage [8], which may influence renal blood flow and reduce excretion from them [9]. Although the mechanism of Dox nephrotoxicity remains unknown, several findings suggest that oxidative stress and inflammation may play a role in the development of Dox-induced renal injury [10,11]. The primary molecular mechanism that explains how Dox causes renal toxicity is reactive oxygen species (ROS) generation. This contributes to an unbalanced redox status, which in turn causes oxidative stress, inflammation, decreased levels of antioxidant, and apoptosis [10]. Nephrologists and oncologists, in general, recently began to support the idea of reducing renal damage during Dox treatment. Recently, interest has increased in the use of herbal medicine for the prevention and/or therapy of renal disorders [12–14].

*Achillea millefolium* (AM), a member of the family *Asteraceae*, is one of these medicinal plants. AM, sometimes called yarrow, has been used in traditional medicine for various ailments. In Yemeni traditional medicine, it is widely utilized. AM is extensively distributed throughout Europe, North America, and Asia. In China, yarrow mainly grows in the northeastern regions of China, Xinjiang, and Inner Mongolia [15]. AM has medicinal properties because of the presence of several different chemical elements, including phenol, flavonoid, sterols, glucosides, terpenoids, alkamides, and coumarins [16]. AM possesses antiinflammatory and antioxidant properties [12,17]. AM was often utilized in traditional medicine to treat conditions such as pain, wounds, hemorrhages, kidney disorders, cancer, cystitis, diabetes mellitus, bleeding disorders, bruises, hemorrhoids, spasmodic diseases, and flatulence and for its gastroprotective, antispasmodic, diuretic, and anxiolytic effects [18,19]. *Achillea biebersteinii*, a close relative of AM, is used widely in Yemen’s folk medicine as an analgesic and antipyretic and to treat diarrhea, flatulence, and liver diseases [20]. The medicinal benefits of AM are recognized globally, and the plant is listed in various national pharmacopoeias [21].

The effects of *Achillea millefolium* extract (AME) against Dox-induced renal injury are unclear. In the present study, the aim was to assess the potential nephroprotective mechanism of AME against Dox-induced nephrotoxicity in rats through oxidative stress and inflammation.

## 2. Materials and methods

### 2.1. Experimental animals

Forty-eight mature male rats weighing between 150 and 250 g were obtained from the Animal House of the Faculty of Veterinary Medicine at Sana’a University in Yemen. The rats were maintained inside cages with controlled humidity (50%) and temperature (22 °C) with 12-h light/dark periods for a week before the experiment. They had unlimited access to tap water and a normal diet was provided. The research was conducted in adherence to ethical protocols and was approved by the institutional ethics committee of the Faculty of Medicine and Health Sciences, Sana’a University (protocol no. 0250-3/3/2022).

### 2.2. Preparation of AME

Aerial parts of AM were gathered from IBB province in Yemen in March 2022 and identified by Dr Ibraheem, Faculty of Science, Sana’a University. A recognized sample of the plant material was deposited at the pharmacy department, Al-Hikma University, Sana’a, with a voucher number (p4-2022). The extract was prepared with minor modifications according to Andleeb et al. [22]. The plant’s raw materials (stems, leaves, and flowers) were rinsed with distilled water, allowed to air dry for 1 week at room temperature in the shade, and blended into a fine powder. Then 200 g of plant powder was macerated with 4 L of 70% ethanol at 25 °C in the dark and dried for 3 days with intermittent shaking. The extract underwent filtration and subsequent evaporation to remove solvent content completely, utilizing a rotary evaporator operating at 40 °C. The purpose of this process was dryness under reduced pressure using a rotary evaporator at 40 °C (RE-52C 1L rotary evaporator, Henan Lanphan Industry Co., Ltd, China). The extract was kept until needed in sterilized dark glass containers at 4 °C. The extract yield percentage (1.46%) was calculated based on initial dry weights. The extract was dissolved in endotoxin-free normal saline (1 mL) at a dosage of 100 or 200 mg/kg/day orally for 28 days [22–24].

### 2.3. Chemicals and reagents

Dox was purchased from Hikma Specialized Pharmaceutical Co., Ltd. (Cairo, Egypt). Formalin was purchased from El-Naser Chemicals (Egypt). Methanol was purchased from Sigma (USA), 0.9% NaCl was obtained from SMSCO (Saudi Arabia), and ethanol was obtained from Merck (Germany).

### 2.4. Experimental design

The rats were allocated randomly into six groups, each comprising eight individuals. Group 1 was the control group. Group 2, the Dox group, received Dox (i.p., 5 mg/kg body weight) specified weekly for 4 consecutive weeks, on days 7, 14, 21, and 28 (one dose per week for 4 weeks equaled totally 20 mg/kg body weight) [25]. Group 3 received AME dissolved in endotoxin-free normal saline (1 mL) at a dosage of 100 mg/kg/day orally for 28 days. Group 4 orally received AME at a dosage of 200 mg/kg/day for 28 days. Group 5 received Dox (5 mg/kg body weight), similar to the second group (Dox group), along with an oral administration of AME at a dosage of 100 mg/kg/day for 28 days. Group 6 received Dox as in the second group (Dox group) plus AME (200 mg/kg/day) orally for 28 days [23–27].

### 2.5. Blood and kidney tissue sampling

Twenty-four hours postexperiment completion, the rats were anesthetized with 2% pentobarbital sodium (0.3 mL/100 g, i.p.). Blood samples from the optic vein and jugular vein were collected in a centrifuge tube. After blood samples were left to clot at 25 °C for 45 min, they were centrifuged for 15 min at 3000 rpm. The serum supernatant was removed and stored at −20 °C for various biochemical tests. Creatinine, uric acid, and urea level were the biochemical measurements made in serum samples from all groups. After that, kidney samples from each rat were collected, weighed, and homogenized to 20% by weight using ice-cooled phosphate buffer (pH 7.4). The renal tissues were homogenized using a glass homogenizer (Universal Lab. Aid MPW-309, Mechanika Precyzyjna, Poland) in 5–10 mL of cold buffer (i.e. 100 mM potassium phosphate, pH 7.0, containing 2 mM EDTA) per gram of tissue. The buffer was centrifuged at 3000 rpm for 15 min at 4 °C, and the supernatant was collected. Furthermore, additional kidney tissue was taken from each group, preserved in 10% natural formalin, and encased in paraffin for histological investigation.

### 2.6. Determination of kidney function parameters

Using commercial kits (BioDiagnostic, Egypt), the concentrations of urea (Cat. No., UR 2110), creatinine (Cat. No. CR 1250), and uric acid (Cat. No. UA 2120) in the serum were detected following the guidelines of the manufacturer using a spectrophotometer (Shimadzu, Japan).

### 2.7. Determination of oxidative stress parameters and antioxidant enzymes

Appropriate kits for testing superoxide dismutase (SOD) (S0101; Beyotime, Shanghai, China), malondialdehyde (MDA) (S0131; Beyotime, Shanghai, China), catalase (S0051; Beyotime, Shanghai, China), glutathione (GSH) (S0052; Beyotime, Shanghai, China), glutathione peroxidase (GPx) (S0056; Beyotime, Shanghai, China), total antioxidant capacity (TAC) (S0116; Beyotime, Shanghai, China), and nitric oxide (NOx) (S0020; Beyotime, Shanghai, China) were adopted for quantification of the above indicators. The SOD, MDA, GSH, GPx, TAC, and NOx measurements can be determined using colorimetric methods and then observed under a microplate reader (1410101; Thermo Fisher Scientific, USA).

### 2.8. Determination of inflammatory markers

The concentrations of the proinflammatory cytokines tumor necrosis factor-alpha (TNF-α), interleukin-1β (IL-1β), and nuclear factor kappa B (NF-κB) in renal tissues were determined using the rat TNF-α kit (RayBio, USA), rat IL-1β kit (Quantikine, R&D Systems, USA), or rat NF-κB kit (Thermo Fisher Scientific) in accordance with the instructions provided by the manufacturer. The optical density (OD) was measured using a microplate reader (BioTek Instruments, Vermont, USA).

### 2.9. Hematoxylin and eosin (H&E) staining

Paraffin sections were dried at 60 °C for 40 min. After dehydration using an ethanol gradient, H&E staining was performed according to the manufacturer’s protocols (G1120; Solarbio, Beijing, China) and then observed under a light microscope (Olympus IX71; Olympus Corp., Tokyo, Japan).

### 2.10. Statistical analysis

Using the Kolmogorov–Smirnov test, the data were found to follow a normal distribution among all groups (p > 0.1). All data are shown as means ± SE. Analysis of variance (ANOVA) was used for comparisons of ≥3 groups, followed by a Bonferroni post hoc test. The differences were considered significant at p < 0.05. GraphPad Prism (version 9.5.0; GraphPad Software, San Diego, CA, USA) was used for statistical analysis and graphing.

## 3. Results

### 3.1. The effects of AME on kidney function parameters in rats treated with Dox

**Figure 1:**
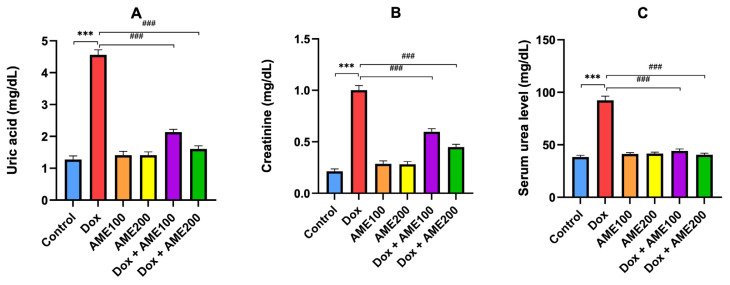
Effects of AME on (A) serum uric acid, (B) creatinine, and (C) serum urea in rats treated with Dox. Results are shown as the mean ± SE (n = 8). ^***^p < 0.001 vs. control group; ^###^p < 0.001 vs. Dox group. [Dox, doxorubicin; AME100, *Achillea millefolium* extract 100 mg/kg; AME200, *Achillea millefolium* extract 200 mg/kg].

Measurements of kidney function parameters, such as serum creatinine, uric acid, and urea, usually used for the detection of kidney injury, were made following the completion of the experiment. The results in Figures 1A–1C indicate that serum uric acid, creatinine, and urea were significantly (p < 0.001) increased in the Dox group in comparison with the control group. Conversely, the rats treated with AME (100 or 200 mg/kg) plus Dox had significantly (p < 0.001) reduced levels of urea, creatinine, and uric acid in comparison with the Dox group. However, the level of reduction was significantly greater in the AME200 plus Dox group than that in the AME100 plus Dox group (p < 0.05). Moreover, the treatment with AME 200 mg/kg plus Dox significantly succeeded in restoring the urea level to the control value.

### 3.2. AME reversed the histopathological changes induced by Dox

**Figure 2:**
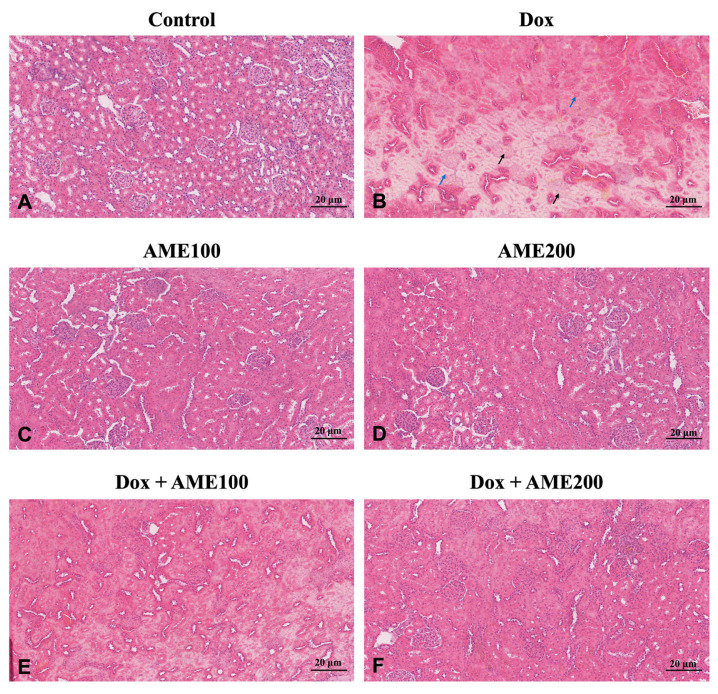
Histopathological evaluation, hematoxylin and eosin staining, A: control group, B: Dox group, C: AME100 group, D: AME200 group, E: Dox + AME100 group, F: Dox + AME100 group, scale bar: 20 μm, black arrows: glomerular degeneration and necrosis, blue arrows: proximal tubule cell degeneration necrosis, proximal tubule epithelial cells edema.

The microscopic examination of the kidney sections in the control group (Figure 2A) showed the normal appearance of renal tissues. The rats given Dox showed glomerular damage and necrosis, tubular dilatation, hypertrophied glomerular capillaries, edema of proximal tubule epithelial cells, and tubular necrosis (Figure 2B), while the microscopic analysis of the kidneys from the AME (AME 100 or 200) treatment group showed a normal appearance of renal tissue (Figures 2C and 2D). The histological analysis of the kidneys of rats given AME 100 plus Dox treatment revealed some glomerulonephritis, but most of the renal tubules had normal structures (Figure 2E). The rats treated with AME 200 plus Dox appeared under the microscope to have quite normal structures, with no evidence of inflammation (Figure 2F).

### 3.3. AME ameliorates Dox-induced nephrotoxicity via inhibition of oxidative stress

**Figure 3:**
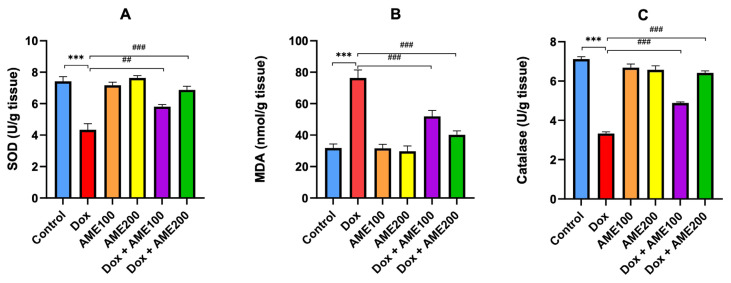
AME ameliorates Dox-induced kidney injury via inhibition of oxidative stress in rats through (A) SOD, (B) renal lipid peroxidase activity as MDA, and (C) catalase activity. Results are shown as the mean ± SE (n = 8). ^***^p <0.001 vs. control group; ^##^p < 0.01 vs. Dox group; ^###^p < 0.001 vs. Dox group. [SOD, superoxide dismutase; MDA, malondialdehyde].

The results presented in Figure 3A indicate that SOD activity was significantly (p < 0.001) decreased in the kidney tissues of rats treated with Dox compared to the control group. This is consistent with previous studies’ findings that Dox-induced nephrotoxicity is associated with oxidative stress [28,29]. In contrast, rats treated with a combination of Dox and AME (100 or 200 mg/kg) showed a significant (p < 0.001) increase in SOD activity in comparison with the Dox group; this improvement was more pronounced (p < 0.05) in the AME (200 mg/kg) plus Dox group. The results of different treatments on lipid peroxidation estimated as MDA are illustrated in Figure 3B. These results revealed that MDA levels were significantly increased (p < 0.001) in the group treated with Dox. In contrast, treatment with AME (100 or 200 mg/kg) plus Dox resulted in significant decreases (p < 0.001) in MDA in comparison with the Dox group, but this reduction was more pronounced (p < 0.01) in the group treated with AME (200 mg/kg).

AME ameliorates Dox-induced toxicity by stimulating antioxidant enzymes such as catalase activity in kidney tissues. The results in Figure 3C indicate that catalase activity was significantly decreased (p < 0.001) in the groups treated with Dox in comparison with the control group. The treatment with AME (100 or 200 mg/kg) plus Dox resulted in significant improvements (p < 0.001) in catalase activity; this improvement was more pronounced in the group treated with AME (200 mg/kg) (p < 0.05).

### 3.4. AME improves GSH, GPx, and TAC and prevents high NOx production in rats treated with Dox

**Figure 4:**
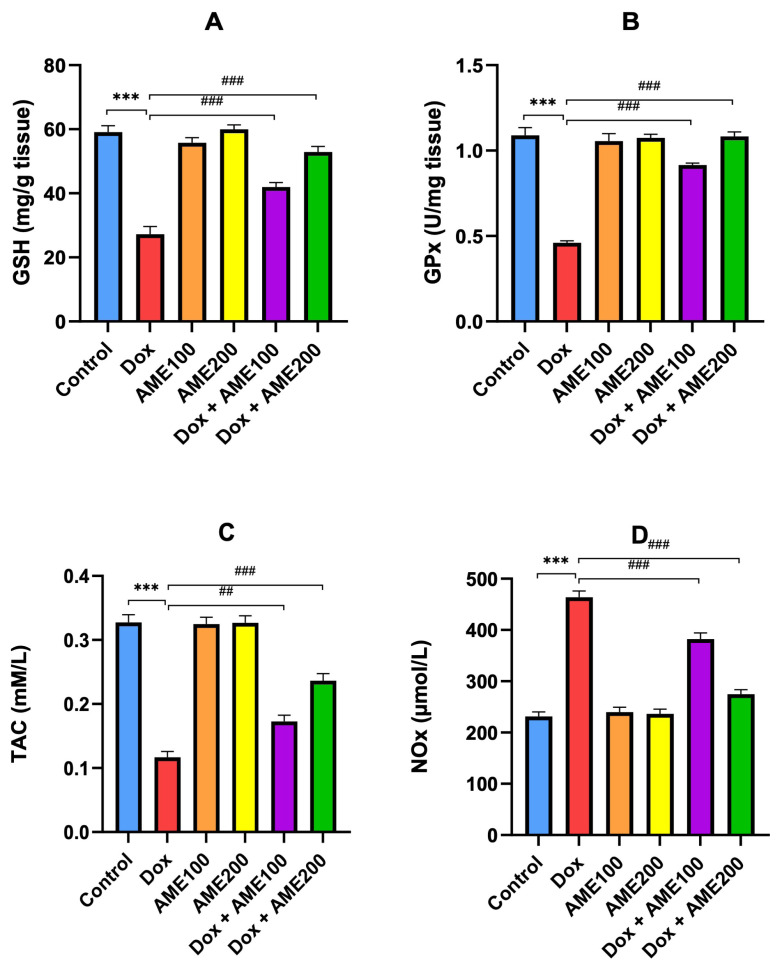
AME attenuated GSH, GPx, and TAC in rats treated with Dox, also AM treatment prevented NOx production during Dox-induced kidney injury in rat. Results are shown as the mean ± SE (n = 8). ^***^p < 0.001 vs. control group; ^##^p < 0.01 vs. Dox group; ^###^p < 0.001 vs. Dox group. [GSH, glutathione; GPx, glutathione peroxidase; TAC, total antioxidant capacity; NOx, nitric oxide].

GSH detoxifies toxic and reactive metabolites and protects cellular systems from their deleterious effects. The results in Figure 4A show that the Dox group exhibited a significant decrease (p < 0.001) in GSH content in comparison with the control, which may be due to its consumption to overcome the excessive free radicals produced by Dox. On the other hand, GSH content was significantly improved (p < 0.001) in rats treated with AME (100 or 200 mg/kg) plus Dox. This improvement was more pronounced in the group treated with AME (200 mg/kg) (p < 0.001). GPx enzymes are essential in protecting the cell from free radical damage, specifically lipid peroxidation. The results in Figure 4B show that the GPx activity in the kidney tissues of rats given Dox was significantly reduced compared to that in the control group (p < 0.001). Treatment with AME (100 or 200 mg/kg) plus Dox resulted in significant improvements in GPx activity compared to the Dox group (p < 0.001). We determined TAC in the kidney. The results for different treatments (Figure 4C) showed that the TAC levels in the group given Dox were significantly less than those of the control group (p < 0.001). The combined treatment of AME (100 or 200 mg/kg) with Dox showed significant improvements (p < 0.001) in TAC in the kidney. Furthermore, this improvement was more pronounced (p < 0.001) in the group treated with AME at 200 mg/kg. The results for different treatments on NOx activity (Figure 4D) showed that NOx was significantly greater in the Dox-treated group. NOx was significantly higher in the group treated with Dox (p < 0.001), while treatment with AME (100 or 200 mg/kg) significantly succeeded (p < 0.001) in protecting against nitric oxide elevation by Dox. This improvement was more pronounced (p < 0.001) in the group treated with AME at 200 mg/kg.

### 3.5. AME ameliorates the inflammation associated with Dox-induced renal injury

**Figure 5:**
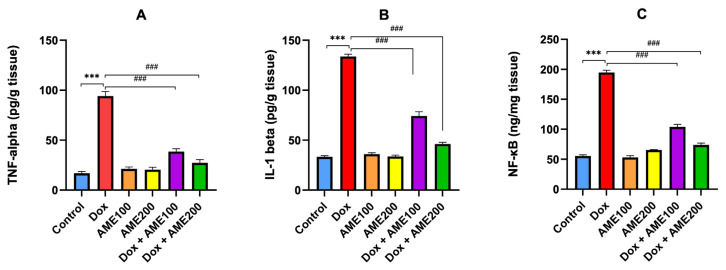
AME ameliorates inflammation in Dox-induced nephrotoxicity by inhibition of inflammatory markers; TNF-α, IL-1β, and NF-κB. Results are shown as the mean ± SE (n = 8). ^***^p < 0.001 vs. control group; ^###^p < 0.001 vs. Dox group. [TNF-α, tumor necrosis factor-alpha; IL-1β, interleukin-1β; NF-κB, nuclear factor kappa B].

AME inhibits proinflammatory cytokines such as NF-κB, IL-1β, and TNF-α produced by Dox treatment (Figures 5A–5C).

When compared to the control group, Dox significantly increased the levels of NF-κB, IL-1β, and TNF-α in the kidneys (p < 0.001). Conversely, when AME (100 or 200 mg/kg) was administered concurrently with Dox, inflammation was considerably (p < 0.001) decreased, along with kidney inflammatory markers, NF-κB, TNF-α, and IL-1β levels. Compared to treatments with AME (100 mg/kg), treatment with AME (200 mg/kg) resulted in a more marked (p < 0.05) reduction in renal contents of NF-κB, TNF-α, and IL-1β.

## 4. Discussion

Dox, a very potent drug with a wide spectrum of anticancer activity, has been in clinical use for over five decades. However, it has many serious adverse effects, including nephrotoxicity [7,30]. The kidney is a highly dynamic and complex organ that contributes to waste elimination, regulates acid–base balance, and helps maintain homeostasis. Toxic substances can impair the function of the kidney [28]. AM has been found to possess several biologically advantageous characteristics, such as antiinflammatory, antioxidative, and anticancer action [16,17,23]. Nevertheless, there has never been any research done on the possible preventive effect of AM against Dox-induced nephrotoxicity.

Reliable markers for kidney damage include creatinine, serum uric acid, and urea levels [31]. The data presented herein showed that Dox administration resulted in a significant rise in the serum levels of uric acid, creatinine, and urea. These elevations indicated successful construction of the Dox-induced nephrotoxicity model. The accumulation of toxic Dox metabolites in nephrons may be the main source of this rise in nephrotoxicity biomarkers, which is consistent with previously published reports showing that Dox adversely affected renal function by significantly raising blood urea and creatinine levels and altering normal renal histology [31]. Interestingly, treatment with AME resulted in a significant decrease in renal function parameters (creatinine, serum urea, and uric acid levels) in the Dox-treated rats, thus offering considerable protection against Dox-induced degenerative changes in the kidney. Additionally, we examined histological changes in the kidney in response to Dox. According to a previous study, Dox therapy altered the morphology of the renal tubules [32]. In the current study, we observed that therapy with AME decreased the pathological injury caused by Dox in kidney tissues, such as edema, vacuolization, and inflammation. These findings revealed that AME might be a novel therapeutic agent for reducing Dox-induced nephrotoxicity, but the mechanism is not comprehensively understood.

It has been established that oxidative stress is the main mechanism of Dox-induced nephrotoxicity [33,34]. AM has medicinal properties because of the presence of a number of different chemical elements, including phenol, flavonoids, sterols, glucosides, terpenoids, alkamides, and coumarins [16]. AM possesses antiinflammatory and antioxidant properties [12,17]. According to Gharibi et al., this plant has demonstrated antioxidant properties and can therefore be used to alleviate complications associated with oxidative stress conditions [35]. Candan et al. [36] investigated the antioxidant and antimicrobial activities of the essential oil and methanol extracts of *Achillea millefolium*. In the present study, we found that MDA concentrations were significantly higher in the Dox-treated group. This indicates that Dox is responsible for lipid peroxidation and oxidative damage to biological molecules. Moreover, pretreatment with AM significantly reduced this effect. Okkay et al. [26] demonstrated that AM substantially reduces MDA levels in chemotherapy-mediated toxicity. GSH plays a pivotal role in controlling the toxic effects of Dox, which may be due to its consumption in an attempt to overcome the excessive free radicals produced by Dox [32]. In the current study, AME markedly increased the expression of GSH, SOD, GPx, and CAT and inhibited NOx in Dox-induced renal injury, indicating that the inhibition of oxidative stress is involved in the protection afforded by AME against Dox-induced nephrotoxicity.

Furthermore, inflammation is considered an important trigger in Dox-induced renal injury [37,38]. The current study revealed that Dox significantly increased the levels of proinflammatory cytokines such as NF-κB, TNF-α, and IL-1β [28,38]. Zolghadri et al. [39] reported that AM reduces inflammation by modulating related signaling pathways. Recent studies have shown that its extract helps alleviate inflammation by lowering levels of inflammatory cytokines like IL-1β. Dorjsembe et al. reported that *Achillea* demonstrated antiinflammatory activity by reducing the release of NOx and prostaglandin E2 (PGE2), as well as decreasing the mRNA expression of inflammatory cytokines in RAW 264.7 macrophages [40]. In a chemotherapeutic toxicity model, an earlier study discovered that AME therapies restored the expression of NF-κB and other mediators of inflammation [26,27]. Our in vivo study demonstrated that AME generated a significant decrease in inflammatory cytokines levels. This implies that inflammation may be the vital mechanism by which AME prevents Dox-induced renal injury. Nonetheless, the concrete mechanism involved still requires detailed elucidation. Toll-like receptors (TLRs), a class of transmembrane receptors, are closely related to inflammation [41]. TLRs can activate NF-κB, directly or indirectly promoting the release of inflammatory factors [42]. Importantly, it has been shown that knockdown of TLRs attenuates Dox-induced cardiotoxicity [43]. Therefore, AME may reduce the activation of TLR/NF-κB pathway and attenuate Dox-induced renal injury.

There are several limitations to the present study. First, the phytochemical content of AME was not examined. AM contains a variety of different bioactive components, including amino acids, monoterpenes, salicylic, terpenoid ketones, folic acid, caffeic acid, and flavonoids [44,45]. Antioxidant activity has been linked to phenolic chemicals. The relatively high concentrations of phenolic chemicals in AM may be responsible for its antioxidant effect [45]. The antioxidant and antiinflammatory activities of AM are also closely related to flavonoids [44]. Second, there are few experimental methods in our study. We will add multiple experimental methods to further enrich the experimental content in our future research and draw more accurate and reliable conclusions from them.

## 5. Conclusion

AME prevents kidney damage induced by Dox by suppressing oxidative stress and enhancing antioxidant-mediated mechanisms, as well as inhibiting proinflammatory cytokines. Overall, our data indicate that AME may be useful as an adjuvant therapy for Dox-induced nephrotoxicity.

## Data Availability

The data that support the findings of this study are available from the corresponding author, upon reasonable request.
